# Seroprevalence of bactericidal antibodies against serogroup B and C Meningococci in a University Hospital

**DOI:** 10.1590/1414-431X20175590

**Published:** 2017-04-20

**Authors:** C.A.C. Gioia, A.P.S. Lemos, M.C.O. Gorla, R. Mendoza-Sassi, B.S. Figueredo, T. Ballester, A. Von Groll, B. Wedig, N.V. Ethur, L. Bragança, P.E.A. Silva, L.G. Milagres

**Affiliations:** 1 Faculdade de Medicina, Campus da Saúde, Universidade Federal do Rio Grande, Rio Grande, RS, Brasil; 2 Departamento de Bacteriologia, Instituto Adolfo Lutz, São Paulo, SP, Brasil; 3 Departamento de Microbiologia, Imunologia e Parasitologia, Universidade do Estado do Rio de Janeiro, Rio de Janeiro, RJ, Brasil

**Keywords:** Neisseria meningitidis, Bactericidal antibodies, Meningococcal carrier

## Abstract

Meningococcus serogroup B (MenB), clonal complex 32 (cc 32), was the Brazilian epidemic strain of meningococcal disease (MD) in the 1990’s. Currently, meningococcus serogroup C (MenC), cc 103, is responsible for most of the cases of the disease in Brazil. The aim of this study was to investigate the seroprevalence of bactericidal antibody (SBA) against representative epidemic strains of MenC, (N753/00 strain, C:23:P1.22,14-6, cc103) and MenB, (Cu385/83 strain, B:4,7:P1.15,19, cc32) in students and employees of a university hospital in the State of Rio Grande do Sul (RS, Brazil). A second MenC strain (N79/96, C:2b:P1.5-2,10, cc 8) was used as a prototype strain of Rio de Janeiro’s outbreak that occurred in the 1990’s. Our previous study showed a 9% rate of asymptomatic carriers in these same individuals. A second goal was to compare the SBA prevalence in meningococcal carriers and non-carriers. Fifty-nine percent of the studied population showed protective levels of SBA titers (log_2_≥2) against at least one of the three strains. About 40% of the individuals had protective levels of SBA against N753/00 and Cu385/83 strains. Nonetheless, only 22% of the individuals showed protective levels against N79/96 strain. Significantly higher antibody levels were seen in carriers compared to non-carriers (P≤0.009). This study showed that, similar to other States in Brazil, a MenC (23:P1.22,14-6, cc103) strain with epidemic potential is circulating in this hospital. Close control by the Epidemiological Surveillance Agency of RS of the number of cases of MD caused by MenC strains in the State is recommended to prevent a new disease outbreak.

## Introduction

Meningococcal disease (MD) is endemic in Brazil, with periodic outbreaks and an average annual incidence rate of 1.4–2.5 cases per 100,000 inhabitants (1). Case fatality rates reach as high as 20% (1). Meningococcus serogroup B (MenB), clonal complex 32 (cc 32) was the Brazilian epidemic strain in the 1990’s. Since 2002, a substantial increase has been observed in the proportion of cases attributed to meningococcus serogroup C (MenC) associated with the sequence type (ST) 103 complex, and it is currently responsible for most of the MD cases in Brazil (1,2). In the State of Rio Grande do Sul (RS), situated in the South of the country, serogroup C became responsible for about 50% of cases of MD only after 2013, followed by serogroup B (about 26%) and serogroup W (∼24%) (2).

Nasopharyngeal carriage of meningococci is common; carriage rates are estimated at ∼10% in Europe, and they rise to >50% in closed or semi-closed institutions, such as universities (3). Smoking, overcrowding, lower socioeconomic status, and male gender are recognized as factors that predict higher rates of meningococcal carriage, particularly in institutions (4,5).

The factors associated with periodic meningococcal outbreaks are not well understood, but they likely include waning population immunity to circulating meningococci, in conjunction with meningococcal carriage and transmission (6,7). Meningococcal carriage in the nasopharynx or oropharynx is typically asymptomatic and transient, lasting weeks to months (6). A small proportion of carriers develop invasive disease (6).

Considerable evidence indicates that complement-mediated serum bactericidal antibody (SBA), induced by naso- or oropharyngeal colonization or vaccination, confers protection against meningococci (8,9).

Few studies have described meningococcal carriage in Brazil (5,10). In contrast to the literature (4,6), a recent study reported high rates of MenC carriage (6.3 and 4.9%) among Brazilian refinery workers (10).

The aim of this study was to investigate the seroprevalence of SBA against representative epidemic strains of MenC, cc 103 (N753/00 strain) and MenB, cc 32 (Cu385/83 strain) in students and employees (n=200) of a university hospital in Rio Grande do Sul. A second MenC strain (N79/96, cc 8) was used in the SBA assay as a prototype strain of Rio de Janeiro’s outbreak occurred in the 1990’s. Our previous study (5) showed a rate of asymptomatic carriers of 9% in this same sample. Therefore, a second goal of this study was to compare the SBA prevalence in meningococcal carriers and non-carriers. Immunoblot studies were done to investigate the specificity of antibodies against protein antigens of meningococcal strains and its association with SBA.

## Material and Methods

### Study population

This study included 200 volunteers from University Hospital Miguel Riet Correia Junior, Fundação Universidade do Rio Grande, RS, Brazil. One hundred volunteers were medical students (20% randomly selected from each year) and the other 100 were hospital workers from different divisions (20% randomly selected from each hospital site). Sample size was calculated based on a prevalence rate of meningococcal carriers of about 30% (±7%) with a 95% confidence interval. Thirty percent of individuals were male. All volunteers usually spent more than 10 h per day in the hospital. Age ranged from 20 to 60 years. Only 10 individuals were smokers. Two subjects reported they had meningitis in childhood but did not know the etiological agent. Oropharyngeal swabs and blood samples were collected during the summer, in December of 2010 and January of 2011 (5). None of the subjects had been vaccinated against meningococcal meningitis before the study. Sera were stored at -70°C. Exclusion criteria included infection symptoms or inflammation, use of antibiotic or immunosuppressive drugs.

### Ethics

This research was approved by the Ethics Committee of the Universidade Federal do Rio Grande do Sul (#102/2011). All project participants provided written informed consent and completed a questionnaire to collect variables considered risk factors for asymptomatic carrier. Individuals were identified by numerical codes.

### Meningococcal strains

MenC strains, N753/00 (C:23:P1.22,14-6, cc 103 and N79/96 (C:2b:P1.5-2,10, cc 8), were representative of two meningococcal disease outbreaks in Brazil. Strain Cu385/83 (B:4,7:P1.19,15) is originally from Cuba (Finlay Institute, Habana), and has the same phenotype and belongs to the same clone (cc 32) as the epidemic Brazilian MenB strain.

### Bactericidal assay

SBA titers against MenC and MenB (corresponding to the above-mentioned genotypes) were measured as previously described, with some modifications (11,12). Briefly, the final reaction mixture contained 25 µL of diluted test serum previously heat-inactivated at 56°C for 30 min, 12.5 µL of human serum that lacked detectable intrinsic bactericidal activity diluted at 1:2 as a complement source, and 12.5 µL of log phase meningococci (about 5×10^3^ CFU/mL). The bactericidal reaction was carried out at 37°C for 30 min (MenB) or 60 min (MenC). The bactericidal titer was defined as the reciprocal serum dilution causing ≥50% killing of the bacteria and is reported as log_2_. The positive control for each assay consisted of post-vaccination human sera with previously determined bactericidal titer. The vaccines utilized were VA-MENGOC-BC^®^ (Instituto Finlay, Cuba) for MenB and conjugated meningococcal C oligosaccharide-CRM197 for MenC (Chiron/Novartis Vaccines, Italy). The complement-independent killing control consisted of heat-inactivated unknown test sera in the presence of heat-inactivated complement and bacteria. The negative control consisted of the complement source in the absence of test serum. Seroprotection was defined as antibody titers ≥4 (log_2_ ≥2) (8,13).

### Immunoblot assay

This assay was applied to characterize the outer membrane proteins (OMP) of meningococcal strains recognized by serum antibodies with or without bactericidal activity. The strains used in the immunoblot were the N753/00 strain (C:23:P1.22,14-6, cc 103), the N79/96 strain (C:2b:P1.5-2,10, cc8) and the Cu385/83 strain (B:4,7:P1.19,15, cc 32). Outer membrane vesicles (OMVs) were prepared by extraction of the wet cell pellet for 2.5 h at 50°C with 5 mL of 0.2 M lithium chloride in a 0.1 M sodium acetate buffer, pH 5.8, per g of cells (14). SDS-PAGE and the detection of antibodies by immunoblot were performed as previously described (14). Each gel (7 by 8 cm) was loaded with 80 μg of OMVs. After electrotransfer at 250 mA for 2 h, each blot was cut into strips with about 3 μg of protein per strip. The strips were blocked (10 mM PBS, pH7.2, plus 3% BSA) and incubated overnight at room temperature with 1:200 dilutions of sera in the presence of 0.15% Empigen BB (EBB, Calbiochem, USA) for partial renaturation of OMPs epitopes (15). All blots were incubated for 2 h with a 1:10,000 dilution of peroxidase-conjugated mouse anti-human IgG+IgM+IgA (total Ig binding) (Kirkegaard & Perry Laboratories, USA) and stained for 15 min with 3-amino-9 ethylcarbazole and hydrogen peroxide. Monoclonal antibodies (mAb) against RmpM (BE12, 1:400 dilution), serosubtype P1.15 (F8-7A2/1H11, 1:5000 dilution), serotype 23 (F129-1G1/1B4, 1/2500 dilution) and serotype 2b (mAb F1-9H10/1B3 1:200 dilution) were used to identify RmpM, PorA and PorB, respectively, and as band intensity controls.

### Statistical analysis

Statistical analyses were performed using the STATA program, version 9.0 (USA). Bactericidal titers are reported as log_2_ mean values and statistical significance was calculated using non-parametric Kruskal-Wallis test. All tests were two-tailed, and P<0.05 was considered to be significant.

## Results

### Distribution of bactericidal antibodies among volunteers

There was a high frequency (59%) of individuals presenting protective levels of SBA titers (log_2_ ≥2) in their blood against at least one of the three meningococcal strains studied. As previously reported, there was also a high prevalence (9%) of carriers of meningococci among these individuals (5). These results are in contrast with other studies describing a low prevalence of carriers and also a low seroprevalence of SBA in university students before an outbreak of MD (4).

A total of 42.5% of the individuals had protective levels of SBA against N753/00 strain (log_2_ mean of 3.65) and 40.5% against Cu385/83 strain (log_2_ mean of 3.3). Nonetheless, only 22% showed protective levels of SBA against N79/96 strain (log_2_ mean of 3.14) (Table 1).

**Table 1:**
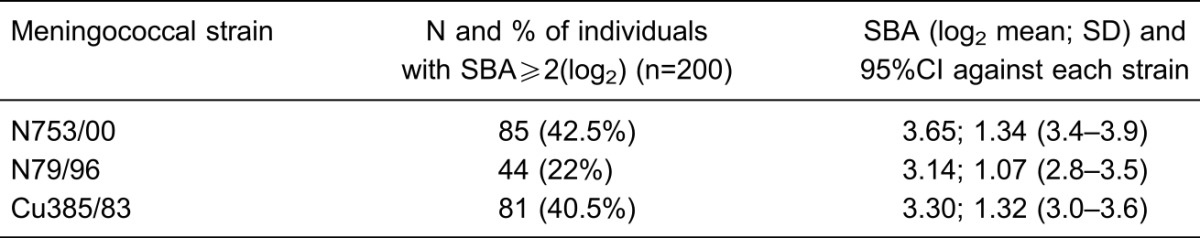
Number (N) and percentage of individuals with protective serum bactericidal antibody (SBA) titers against meningococcal strains.

### Association between meningococcal carriage and SBA

Significantly higher antibody levels were seen against the three meningococcal strains in carriers (n=18) compared to non-carriers (n=182) (P≤0.009, Table 2). Despite the difference in sample size, meningococcal carriers had a mean of two times more antibodies than meningococcal non-carriers. In comparison with N753/00 strain, lower bactericidal antibody titers were detected against N79/96 strain for both meningococcal carriers (P=0.03) and non-carriers (P=0.001).

**Table 2:**
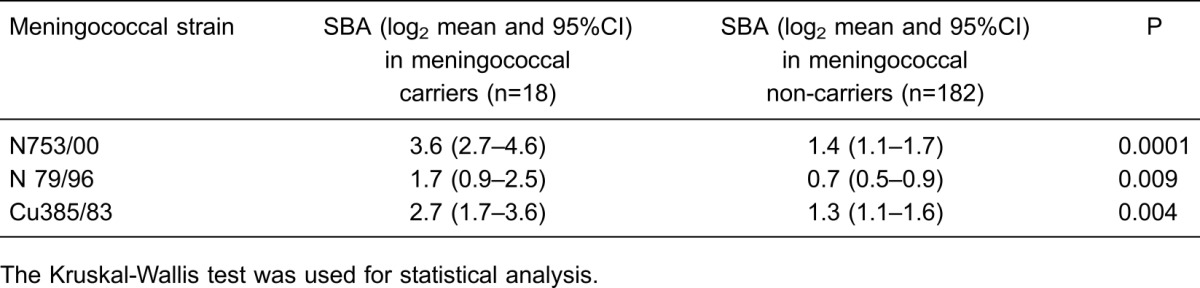
Serum bactericidal antibody (SBA) against meningococcus serogroup C and meningococcus serogroup B strains in carriers and non-carriers of Neisseria meningitidis.

As shown in Table 3, about 78 and 83% of carriers had protective titers of SBA against MenC (N753/00) and MenB (Cu385/83) strains. In comparison, a much lower (about 37.5%, P≤0.005) frequency of non-carriers had SBA against those two strains. N79/96 strain was recognized (log_2_ SBA≥2) by approximately 56% of carriers and only by 19% of non-carriers (P=0.02).

**Table 3:**
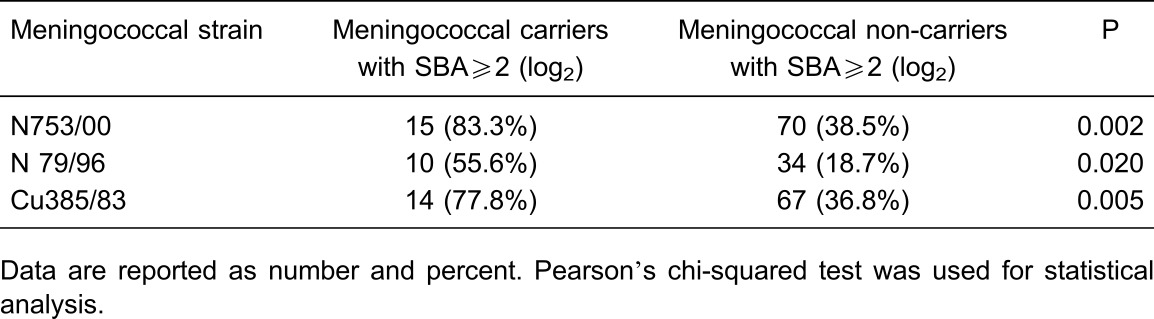
Frequency of carriers (n=18) and non-carriers (n=182) with protective levels of serum bactericidal antibodies (SBA) against meningococcal strains.

Among the 18 meningococcal carriers, two (11%) of them did not have a protective level of SBA against any strain of this study. Among non-carriers, 45% of the 182 individuals had no protective levels of SBA.

Table 4 shows that meningococcal carriers recognized the MenB and MenC strains at a frequency three to four times higher (P=0.09) than non-carriers, suggesting the recognition of common antigens not related to the capsule.

**Table 4:**
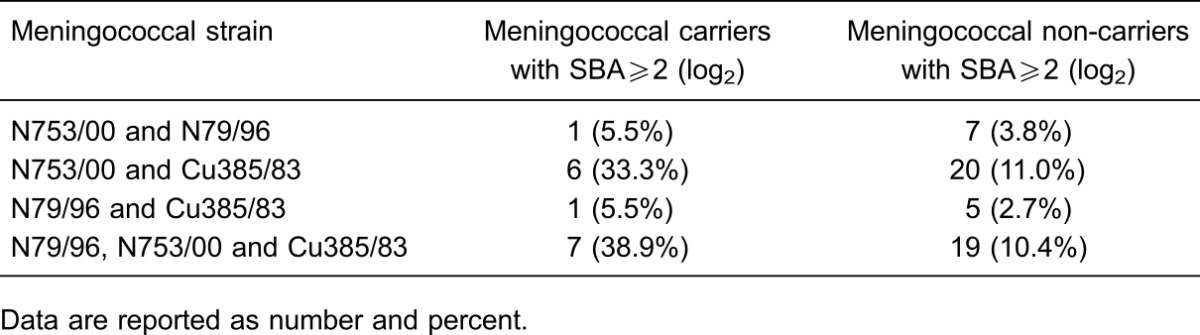
Frequency of carriers (n=18) and non-carriers (n=182) with protective levels of serum bactericidal antibodies (SBA) against more than one meningococcal strain.

### Immunoblot

**Figure 1:**
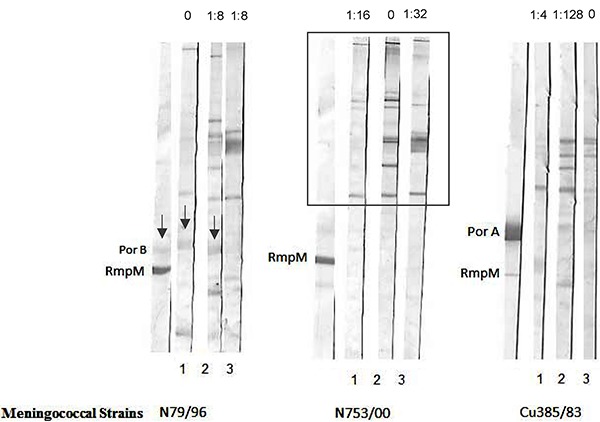
Immunoblot reaction with outer membrane vesicles from meningococcus serogroup C strains N79/96 and N753/00, and meningococcus serogroup B strain Cu385/83. Strips 1, 2 and 3 represent serum samples from carriers. Serum bactericidal antibody titer is shown on the top of each strip (log_2_). Monoclonal antibodies against PorB (F1-9H10/1B3, weak reaction), PorA (F8-7A2/1H11) and RmpM (BE12) were used as control markers and as positive controls in the first strip.

Figure 1 shows the total Ig binding of serum from 3 volunteers representative of 14 serum samples analyzed. Three of 14 individuals recognized the PorB protein of N79/96 strain (see arrows in strips 1 and 2 for two individuals). One individual showed Ig binding to PorB of N753/00 (data not shown). There was no association between PorB recognition and bactericidal activity as exemplified by serum in strip 1 which recognized PorB but had no SBA titer. In contrast, serum in strip 2, which also recognized PorB and other proteins, showed a SBA titer of 1:8 (log_2_=3). Only one individual showed a suggestive binding to PorA protein (data not shown), the main OMP associated with bactericidal antibodies in the literature (16). As can be seen in Figure 1, most of the serum samples recognized proteins of high molecular weight in the three strains studied (delineated by a square in N753/00 strain). The same pattern was observed for sera from non-carriers (data not shown). In summary, there was no association between the recognition of a certain OMP and SBA titer, as we previously described for vaccinees (MenB Cuban vaccine) and PorA (16).

## Discussion

This study showed an important seroprevalence of SBA to MenC and MenB strains in a university hospital. Our data showed major differences between the two MenC strains used as bactericidal antibody target strains. We do not know the reason for these different strain sensitivities to SBA. It could be related to variable antigenicity of the strains but it could also be a consequence of the current epidemiologic aspects of the disease in RS. Strain N753/00 belongs to cc 103, the current epidemic clone in Brazil. MenC cc 103 has caused epidemics in two refineries in São Paulo, located in the southeast of the country, and was also described as the cause of an outbreak in the northeast of the country (10,17). Strain N79/96 cc 8 is a prototype strain of Rio de Janeiro’s outbreak occurred in the 1990’s (18). Of note, since 2002, serogroup C prevalence has overtaken serogroup B cases in most regions of Brazil. However, only after 2013 MenC was prevalent in the south region of the country (2). The high prevalence of MenB and MenC (probably cc 103) in RS in the last years might have contributed to the reported similar proportion of individuals with protective levels of SBA titers against the MenB and MenC (N753/00) strains.

These results suggest the circulation of MenC strain (cc 103) and MenB strain in the hospital. Interestingly, our previous study (5) showed no isolation of MenC strains among carriers after only one oropharyngeal swab. Nonetheless, the relatively high seroprevalence of SBA among the university hospital’s population indicates exposure to and acquisition of MenC (especially the cc 103) and MenB strains. The results of immunoblot reaction indicate that in opposition to responders to MenB OMVs vaccines who recognize mainly PorA and PorB proteins (16), natural immunity is more diversified, and for unknown reasons those proteins are less immunogenic or less exposed to the immune system during colonization. The same seems to be true for MenC strain since most of the sera reacted with high molecular weight proteins.

The absence of protective antibodies in many subjects of the study and the close contact with asymptomatic meningococcal carriers circulating in the hospital indicates the necessity of adopting preventive strategies to avoid meningococcal outbreaks.

Serial carriage and seroprevalence surveys in a community sample could provide better insight if the hospital seroprevalence reported here represents the regional seroprevalence. Future studies will be useful to link the duration of natural immunity with the appearance of new meningococcal clones causing disease. These studies are also very important before the introduction of new vaccines.
